# Establishment of a VSV-Based Pseudovirus Platform for In Vitro and In Vivo Evaluation of Nipah Vaccine-Induced Neutralizing Responses

**DOI:** 10.3390/v17111429

**Published:** 2025-10-28

**Authors:** Seong Eun Bae, Minhyuk Yoon, Younghye Moon, Min Jung Kim, Jeong-In Kim, Kee-Jong Hong, Jae-Ouk Kim

**Affiliations:** 1 Molecular Immunology, Science Unit, International Vaccine Institute, Seoul 08826, Republic of Korea; 2 Department of Bio-Medical Sciences, Gachon University, GAIST, Incheon 21936, Republic of Korea; 3 Department of Microbiology, Gachon University College of Medicine, Incheon 21936, Republic of Korea; 4 Lee Gil Ya Cancer and Diabetes Institute, Gachon University, Incheon 21999, Republic of Korea

**Keywords:** virus, zoonotic pathogen, vaccine-induced immunity, preclinical evaluation, pseudovirus, immunoprecipitation, neutralization, bioluminescent imaging

## Abstract

The Nipah virus (NiV) is a zoonotic pathogen characterized by high fatality rates and pandemic potential, whereby there is an urgent need for developing safe and effective vaccines. However, the evaluation of NiV vaccine-induced immunity is hindered by the requirement of Biosafety Level-4 (BSL-4) containment. In this study, we developed a recombinant vesicular stomatitis virus (rVSV)-based pseudovirus-expressing NiV fusion (F) and attachment (G) glycoproteins using a luciferase reporter gene for bioluminescence detection. This pseudovirus was optimized for production in BHK-21 (WI-2) cells, and simultaneous incorporation of NiV-F and NiV-G onto the surface of the pseudotyped virus was confirmed via immunoprecipitation and Western blotting. We evaluated our pseudovirus-based neutralization assay using NiV-F-immunized mouse sera and a commercial anti-NiV-G antibody, confirming robust neutralization by the latter. To establish a BSL-2-compatible model for evaluating in vivo protective efficacy, we performed in vivo imaging, which revealed a marked reduction in the luminescence signal in NiV-G-immunized mice compared to naïve controls, indicating vaccine-induced protection. Our study established an integrated in vitro and in vivo pseudovirus platform using rVSV that enables safe, quantitative, and BSL-2-compatible evaluation of NiV vaccine candidates. This model offers a valuable tool for preclinical screening of vaccine-induced neutralizing antibody responses and protective efficacy.

## 1. Introduction

The Nipah virus (NiV), a member of genus *Henipavirus* within *Paramyxoviridae*, is an emerging zoonotic pathogen capable of causing severe encephalitis and respiratory illness in humans. Since its initial outbreak in Malaysia in 1998, NiV has caused recurrent outbreaks in South and Southeast Asia, with fatality rates exceeding 70% in some regions. More recently, several significant NiV outbreaks have occurred in South Asia, including the devastating 2018 Kerala outbreak with 19 cases and 17 deaths, the 2023 Bangladesh outbreak resulting in 11 cases and 8 fatalities, and the ongoing 2025 Kerala outbreak with 4 confirmed cases and 2 deaths [1,2,3]. These events demonstrate the persistent public health threat posed by NiV through sporadic zoonotic spillover from bat reservoirs and subsequent human-to-human transmission. Owing to its high pathogenicity, human-to-human transmission potential, and lack of licensed vaccines or therapeutics, NiV has been designated as a priority pathogen by the World Health Organization.

Effective vaccine development against NiV requires a reliable assessment of vaccine-induced neutralizing antibody responses and protective efficacy. However, direct evaluation using live NiV poses substantial challenges owing to BSL-4 containment requirements, limited accessibility to facilities, and biosafety risks. As a result, alternative approaches using pseudotyped viral systems have gained increasing attention as safe, scalable, and informative tools for the preclinical evaluation of vaccine candidates.

Recombinant vesicular stomatitis virus (rVSV)-based pseudoviruses represent a versatile platform for mimicking the entry of high-risk viruses. By incorporating the envelope glycoproteins of target pathogens along with a luciferase reporter gene, rVSV pseudoviruses allow for quantitative infectivity measurements under BSL-2 conditions. Moreover, when coupled with in vivo imaging, these pseudoviruses offer a non-lethal method for assessing real-time infection dynamics and vaccine-induced protection in animal models [4,5].

Although lentivirus-based pseudoviruses have been previously used to evaluate NiV vaccine candidates and monoclonal antibodies in vivo [6], the use of VSV-based pseudoviruses for in vivo bioluminescent imaging of vaccine-induced protection in mice has not been well established.

In this study, we engineered an rVSV-based pseudovirus-expressing the NiV-F and NiV-G glycoproteins and evaluated its usefulness in both in vitro neutralization assays and in vivo protection studies. We optimized pseudovirus production, verified the co-expression of NiV-F/G on the viral surface, and demonstrated neutralization of the pseudovirus produced using both immunized mouse sera and commercial antibodies. Importantly, we established a mouse challenge model using this pseudovirus and successfully visualized the vaccine-mediated inhibition of infection in vivo through bioluminescent imaging. This novel, integrated pseudovirus system provides a safe and robust platform for the comprehensive preclinical evaluation of NiV vaccine candidates, encompassing both humoral immune responses and protective efficacy. Furthermore, this system may serve as a versatile model not only for NiV but also for other emerging BSL-4 pathogens, thereby allowing for ready preclinical evaluation under BSL-2 conditions.

## 2. Materials and Methods

### 2.1. Cells

Vero76 (Korean Cell Line Bank, Cat. No. KCLB-21587, Seoul, Korea) cells were maintained in Dulbecco’s modified eagle medium (DMEM, Gibco™, Cat. No.11995-065, Boston, MA, USA) supplemented with 10% fetal bovine serum (FBS, Gibco, Cat. No.26140-079, Boston, MA, USA) and 1× penicillin–streptomycin (100× P/S, Gibco™, Cat. No.15140-122, Boston, MA, USA) in a 5% CO_2_ environment at 37 °C. BHK-21 (WI2) (Kerafast, Cat. No. EH1011, Boston, MA, USA) cells were maintained at 37 °C in DMEM supplemented with 5% FBS and 1% P/S in an 8% CO_2_ environment.

### 2.2. Generation of NiV Fusion Glycoprotein-Specific Antisera in Mice

Five-week-old BALB/c mice were purchased from Orient Bio (Seongnam, Korea) and acclimated to the animal facility for 1 week prior to experimentation. All procedures were approved by the Institutional Animal Care and Use Committee (IACUC No. 2017–008). Mice were immunized intramuscularly at 2-week intervals for administration of four doses. Each dose consisted of 1 μg of recombinant Nipah virus fusion glycoprotein F (NiV-F, Recombinant Nipah Virus Glycoprotein F (UniProt Q9IH63; Fc Chimera, Abcam, Cat. No. Ab256443, Cambridge, UK)) mixed with an aluminum hydroxide-based adjuvant (Imject Alum, Thermo Fisher, Cat. No. 77161, Waltham, MA, USA) at 1/4 of the total volume, in a final volume of 50 μL per injection. The injections were alternated between the left and right hind limbs for each immunization. 1 week after the fourth immunization, blood samples were collected to obtain serum from immunized mice. The resulting serum (NiV F-immunized mouse serum) was used for Western blotting (WB) and neutralization assays.

### 2.3. Production of the Vesicular Stomatitis Virus (VSV)-Based Pseudovirus

The NiV-F and NiV-G genes of the Malaysian strain (GenBank accession no. AAK50544.1 and AAK50545.1, respectively), were codon-optimized, synthesized using GenScript (Piscataway, NJ, USA), and cloned into the mammalian expression vector pCAGGS-Kan (Kerafast, Cat. No. EH1017, Boston, MA, USA). Subsequently, BHK-21 (WI-2) cells were transfected with a total of 16 μg of DNA at various NiV F to NiV G ratios. For a ratio of F:G = 1:2, 5.33 μg of NiV-F and 10.66 μg of NiV-G were transfected per 100 mm dish using Lipofectamine 2000 (Invitrogen, Cat. No. 11668019, Frederick, MD, USA) according to manufacturer instructions. Then, at 24 h post-transfection, cells were infected with VSV-G*ΔG-Luciferase (Kerafast, Cat. No. EH1020-PM; Boston, MA, USA) at a multiplicity of infection (MOI) factor of 4 [7]. This VSV-ΔG system harbors a deletion of the native VSV G glycoprotein, rendering a replication-incompetent virus that is dependent on heterologous envelope proteins for a single round of infection. During viral assembly, the codon-optimized NiV-F and NiV-G expressed in transfected cells were incorporated into budding virions, generating pseudotyped particles suitable for entry and neutralization assays under BSL-2 conditions. Pseudovirus production was performed as previously described, with minor modifications to optimize NiV-F/G incorporation. Our protocol closely followed that of Loomis et al. [8] and Moon et al. [9], who also used VSV-ΔG backbones and codon-optimized glycoprotein expression to generate high-titer henipavirus pseudotypes for neutralization assays. Then, after 1 h, the cells were washed and the medium was replaced with fresh DMEM containing 5% FBS and incubated at 37 °C in an 8% CO_2_ environment for an additional 24 h. Culture supernatants containing the rVSV-NiV-F/G pseudovirus were harvested, filtered through a 0.45 μm filter (Nalgene, Cat. No. 165-0045, Frederick, MD, USA) and stored at −80 °C until further use. Images of the co-expression of both NiV-F and NiV-G during pseudovirus production were captured using a Nikon ECLIPSE Ts2 (Tokyo, Japan) with a 20× phase objective.

### 2.4. Verification of F/G Co-Expression on the Pseudovirus via Immunoprecipitation (IP) and Western Blotting for F/G Detection

The harvested rVSV-NiV-F/G supernatants were prepared and loaded onto 12% iodixanol cushion (Optiprep^TM^, Serumwerk Bernburg AG, Cat. No.1114542, Bernburg, Germany) in a polypropylene centrifuge tube (Beckman Coulter, Cat. No. 326819, Brea, CA, USA). Samples were ultracentrifuged at 154,000× *g* for 2 h at 10 °C using an Optima XE-100 (Beckman Coulter, Brea, CA, USA) equipped with SW55Ti rotor (Beckman Coulter, Brea, CA, USA). After ultracentrifugation, the fractions were divided into an upper, a middle, and a lower layer.

The lower fraction was incubated with rabbit anti-NiV glycoprotein antibody, IgG fraction (1:50, Alpha Diagnostic International, Cat. No. NIV11-S, San Antonio, Texas, USA), and protein A agarose beads (Millipore, Cat No. IP02-1.5MLCN, Boston, MA, USA). Normal rabbit IgG (Sino Biological, Cat. No. CR1, Beijing, China) was used as a negative control for immunoprecipitation (IP).

After washing, pellets were resolved by 4–20% gradient SDS-PAGE (Bio-Rad, Cat. No. 4561095; Brea, CA, USA) and transferred to a polyvinylidene fluoride membrane (Bio-Rad, Cat. No. 162-0239, Brea, CA, USA). Then, this membrane was incubated with NiV F-immunized mouse serum (1:500), anti-VSV-M (23H12) mouse IgG2a (1:2000; absolute antibody, Cat. No. Ab01404-2.0, Cleveland, UK). The blots were further incubated in blocking buffer containing horseradish peroxidase (HRP)-conjugated goat anti-mouse IgG (1:5000, Southern Biotech, Cat. No. 1030-05, Birmingham, AL), and detection was performed with ECL reagent using the Amersham Imager 680 (GE Healthcare, Chicago, IL, USA).

### 2.5. Pseudovirus-Based Neutralization Assay (PBNA)

Vero76 cells were seeded onto a black, flat-bottomed 96-well assay plate (Corning, Cat. No. 3916, Phoenix, Phoenix, AZ, USA) and incubated at 37 °C with 5% CO_2_ for 24 h. Mouse and rabbit serum samples were serially diluted 2-fold starting from 1/10 to 1/1280 and incubated with rVSV-NiV-F/G diluted to 200 TCID_50_ in DMEM containing 10% FBS and 1× P/S at room temperature (20–25 °C) for 1 h. The infectious titer of the pseudovirus (expressed as TCID_50_) was determined as described in Section 2.6. A rabbit anti-NiV glycoprotein antibody (Alpha Diagnostic International, Cat. No. NIV11-S, San Antonio, TX, USA), raised against recombinant full-length NiV G glycoprotein (UniProt Q9IH62), was used as the positive control and diluted 3-fold starting from 1/20 to 1/43740. Normal rabbit control IgG (Sino Biological Cat. No. CR1 HC13MA0602, Beijing, China) was used as a negative control.

After incubation, the cell culture medium was removed from the Vero76 cells, and the serum–pseudovirus mixture was added to the cells. The plates were then incubated at 37 °C with 5% CO_2_ for 24 h, following which the mixture was removed, and the Vero76 cells were lysed with 50 μL of ONE-Glo™ EX Reagent (Promega, Cat. No. E8130, Madison, WI, USA) on a Microplate Shaker (Jeio Tech, Cat. No. CPS-350, Daejeon, Korea) at 600 rpm for 5 min. Bioluminescence was measured in relative light units (RLUs) at 570 nm using a SpectraMax L Microplate Reader (Molecular Devices, San Jose, CA, USA). Finally, the percentage of neutralization was calculated and the half-maximal inhibitory concentration (IC_50_) was determined using the GraphPad Prism 10 software [10].

### 2.6. Determination of Infectious Concentration of the Experimental Pseudovirus

Vero76 cells were seeded into 96-well assay plates and cultured at 37 °C in a 5% CO_2_ atmosphere. Then, 24 h after seeding, the culture medium was removed. Purified rVSV-NiV-F/G was obtained from the lower fraction collected after ultracentrifugation under the conditions described in Section 2.4. The purified pseudovirus was diluted five-fold (four replicates for each concentration) in fresh culture medium and added to each well to induce pseudovirus infection. Lastly, 24 h after infection, the bioluminescence of each well was measured as described above.

To determine whether a well was infected, the average of the luminescence signal of cells to which no pseudovirus was added was used as a negative control, and wells in which the recorded luminescence signal was more than three times that of the “negative” control were determined as “positive” wells. Based on the “negative” and “positive” results of each well, the tissue culture infectious dose 50% (TCID_50_) was calculated by the method of Reed and Muench [11].

### 2.7. In Vivo Evaluation of Protective Efficacy for NiV Vaccine Candidates

Six-week-old BALB/c mice were purchased from Orient Bio (Seongnam, Korea) and acclimated to the animal facility for 1 week. After the acclimation period, the mice were randomly divided into two groups (four mice per group): PBS (control group) and NiV-G (vaccination group). Purified recombinant NiV-G protein (183-602 aa) was kindly provided from the laboratory of Dae Gwin Jeong at the Korea Research Institute of Bioscience and Biotechnology (KRIBB) and used for vaccination. Mice in each group received either the vaccine (10 μg/mouse) formulated with aluminum hydroxide (Alhydrogel adjuvant 2%; InvivoGen, Toulouse, France) or PBS (A total of eight mice were used, N = 1 for each concentration treatment for each of the two experimental groups). The vaccine was injected intramuscularly twice with a 2-week interval between injections. Two weeks after the 2nd vaccination, purified rVSV-NiV-F/G was intraperitoneally administered to each mouse at the indicated concentrations; 2, 1, 0.5, and 0.25 TCID_50_. The pseudovirus did not cause any suffering to the experimental subjects and no unexpected adverse events were observed.

According to a previous in vivo imaging study using VSV-based pseudoviruses [5], four hours after NiV-F/G-pseudovirus infection, in vivo bioluminescence imaging was conducted using an in vivo imaging system (Spectral Instruments Imaging, Tucson, AZ, USA) at the Core Facility for Cell-to-In Vivo Imaging of the Lee Gil Ya Cancer and Diabetes Institute of Gachon University. Mice were administered an intraperitoneal injection of RediJect D-luciferin (150 μL/mouse; 150 mg/kg body weight; Perkin Elmer, Waltham, MD, USA). Then, the mice were anesthetized in an induction chamber with 3% isoflurane (Hana Pharm Co., Ltd., Hwaseong, Korea) at a flow rate of 0.5 L/min. Bioluminescent signals were detected 10 min later using the Aura Image Analysis Software (Version 2.2.1.1, Spectral Instruments Imaging, Tucson, AZ, USA). Relative bioluminescence signals were measured in photons per section per square centimeter per steradian (photons/sec/cm^2^/sr) and are shown as RLU. In vivo experiments were performed using a minimal number of animals to demonstrate the feasibility of applying pseudoviruses to animal testing. No data were excluded from the dataset obtained from each experiment. Animal experiments were conducted with approval from the Center of Animal Care and Use of the Lee Gil Ya Cancer and Diabetes Institute of Gachon University (LCDI-2023-0144).

## 3. Results

### 3.1. Optimization of NiV-F/G-Pseudotyped VSV Production

**Figure 1:**
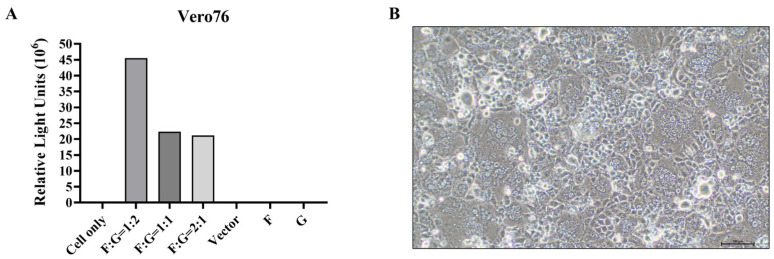
Optimization of Nipah F/G-pseudotyped rVSV production and transduction efficiency. (**A**): Transduction of Vero76 target cells with rVSV pseudotyped with Nipah virus F and G glycoproteins produced in BHK-21 (WI-2) cells using various plasmid F:G ratios. Bioluminescence was measured at 570 nm as relative light units (RLUs), 24 h post-transduction. (**B**): Cell morphology of BHK-21 (WI-2) cells after transfection with Nipah F and G plasmids at a 1:2 ratio. Images were captured to assess cytopathic effects.

To optimize the conditions for the production of rVSV-NiV-F/G with the highest infectivity, we evaluated three different plasmid ratios of NiV-F to NiV-G (1:2, 1:1, and 2:1) along with the following control groups: pCAGGS vector-only, NiV-F only, and NiV-G only. Because our VSV-based pseudovirus system contains a luciferase reporter gene, we compared the infectivity of the produced pseudoviruses by measuring the RLU in Vero76 cells. The pseudovirus produced using a 1:2 F:G ratio showed the highest infectivity, yielding an RLU of approximately 4.5 × 10^7^, nearly twice that observed when the 1:1 or 2:1 ratio was used (Figure 1A). Pseudoviruses expressing only NiV-F or NiV-G showed low RLU levels similar to those in the cell- and vector-only groups (Figure 1A).

Syncytia formation during the production of the pseudovirus was also observed. Co-transfection with NiV-F and NiV-G resulted in pronounced syncytia formation in pCAGGS-NiV-F/G-transfected cells, whereas the control groups (vector only, F only, and G only) did not exhibit any syncytia formation (Figure 1B). These observations suggest that the co-expression of both NiV-F and NiV-G is required for effective production of the pseudovirus.

### 3.2. Purification and Infectivity Assessment of rVSV-NiV-F/G Particles Following Ultracentrifugation

**Figure 2:**
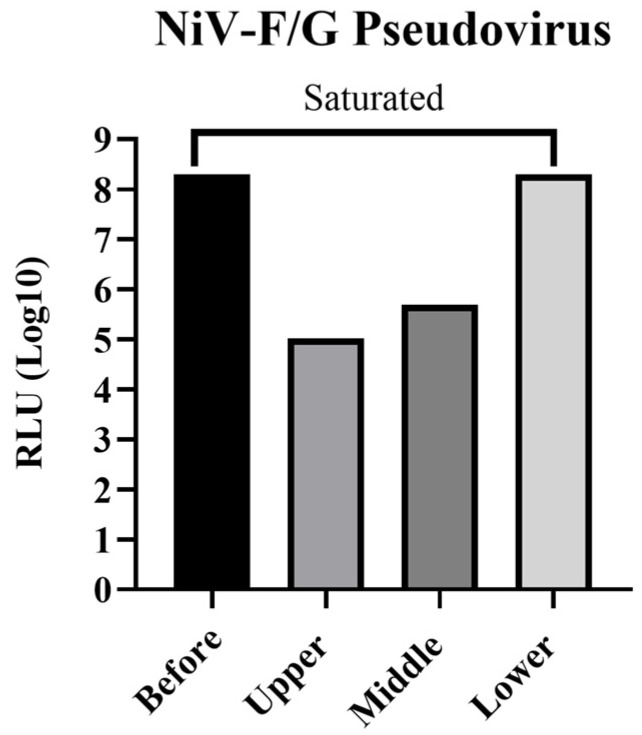
Assessment of transduction efficiency of NiV F/G-pseudotyped rVSV after ultracentrifugation. Virus-containing supernatants were ultracentrifuged at 154,000× *g* for 2 h at 10 °C and fractionated into an upper, a middle, and a lower layer. The transduction efficiency of each fraction was evaluated using a luciferase reporter assay in Vero76 cells. Bioluminescence was measured as RLU at 570 nm, at 24 h post-transduction.

To assess the distribution of infectious rVSV-NiV-F/G particles, virus-containing supernatants were subjected to ultracentrifugation and fractionated into the upper, the middle, and the lower layers. The infectivity test in Vero76 cells revealed that the lower fraction showed the highest RLU values, indicating that functional pseudoviruses were predominantly enriched in denser layers (Figure 2). In contrast, minimal infectivity was detected in the upper and middle layers, suggesting a limited viral content. Visualization of the RLU values on logarithmic scales further confirmed substantial differences in infectivity among fractions, with the most concentrated ones approaching signal saturation. These findings demonstrate that the effective recovery of infectious pseudovirus particles can be achieved by selecting the appropriate post-ultracentrifugation fractions.

### 3.3. Verification of NiV-F and NiV-G Co-Expression in Pseudotyped Viral Particles

**Figure 3:**
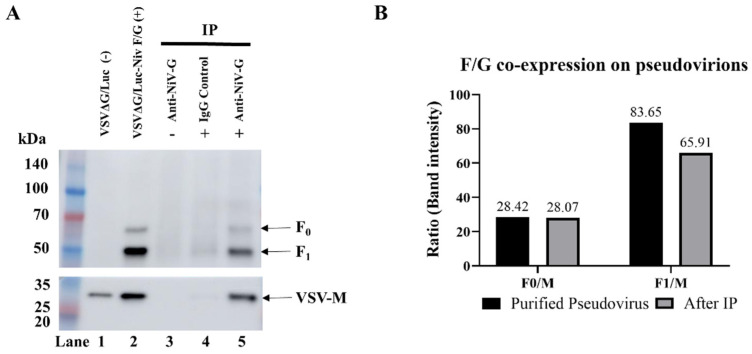
Co-expression of NiV F and G glycoprotein on NiV F/G-pseudotyped rVSV virions. (**A**): Immunoprecipitation (IP) was performed using anti-NiV G antibody (Rabbit pAb) to verify co-expression of F and G glycoproteins in purified NiV F/G-pseudotyped rVSV particles. Membranes were probed with anti-NiV F (1:500) and anti-VSV-M (1:2000), followed by HRP-conjugated goat anti-mouse IgG (1:5000). Western blot analysis showing NiV F (upper panel) and VSV-M (lower panel). Lane 1: purified rVSV lacking F/G expression; lane 2: purified NiV F/G-pseudotyped rVSV; lane 3: IP of rVSV (no F/G) with anti-G antibody; lane 4: IP of NiV F/G-pseudotyped rVSV using isotype-matched normal rabbit control IgG as a negative control; lane 5: IP of NiV F/G-pseudotyped rVSV with anti-G antibody. F_0_: precursor form of NiV F; F_1_, cleaved form. (**B**): Band intensity ratio of F_0_ and F_1_ relative to VSV-M.

To investigate the co-expression of NiV-F and NiV-G in pseudovirus particles, we purified rVSV-NiV-F/G by ultracentrifugation. When these purified pseudoviruses were analyzed by WB to assess the expression of NiV-F, two bands, at ~65 kDa (NiV-F0, precursor form) and ~50 kDa (NiV-F1, cleaved form), were detected along with the VSV-M band (Figure 3A, Lane 2). In contrast, purified rVSVΔG/Luc, which did not express NiV-F/G, displayed a single band at approximately 29 kDa, which corresponded to VSV-M, thus confirming the absence of NiV-F and NiV-G (Figure 3A, Lane 1).

To confirm that NiV-F and NiV-G were incorporated into the same viral particles, we performed IP using an anti-NiV-G antibody followed by WB detection of the purified pseudotyped NiV-F particles. As shown in Figure 3A, lane 3 (IP-purified rVSVΔG/Luc with anti-NiV-G) showed no bands, confirming that rVSVΔG/Luc particles contained neither NiV-G nor NiV-F. In turn, lane 4 (IP-purified rVSV-NiV-F/G with an IgG control antibody) showed no bands either, indicating the absence of non-specific binding. In contrast, lane 5 (IP-purified rVSV-NiV-F/G with anti-NiV-G) showed bands corresponding to NiV-F0 (~65 kDa), NiV-F1 (~50 kDa), and another one corresponding to VSV-M (~29 kDa), thus confirming that NiV-F and NiV-G were co-expressed within the same pseudotyped viral particles.

We further assessed NiV-F incorporation by comparing the F0/VSV-M and F1/VSV-M ratios before and after IP (Figure 3B). The F0/VSV-M ratio remained stable regardless of IP (28.42 and 28.07, respectively), but the F1/VSV-M ratio was slightly lower in the particles immunoprecipitated with NiV-G (65.91) than in the purified pseudovirus (83.65), suggesting that the IP process with NiV-G may have preferentially retained the NiV-F precursor (F0) relative to the mature F1 form. These results confirmed the successful co-expression of NiV-F and NiV-G on the same pseudotyped particles.

### 3.4. Applicability of the Pseudovirus-Based Neutralization Assay (PBNA) to Neutralizing Antibody Evaluation

**Figure 4:**
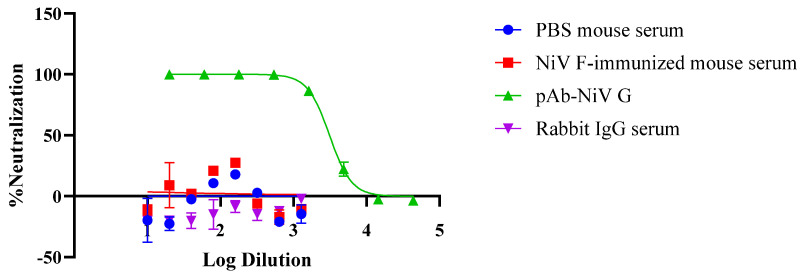
Neutralization of NiV F/G-pseudotyped rVSV by immunized mouse serum and anti-NiV G pAb. Mouse sera were serially diluted 2-fold from 1:10 to 1:1280. Commercial polyclonal anti-NiV G antibody (pAb-NiV G; NIV11-S) was diluted 3-fold from 1:20 to 1:43,740 and used as a positive control. The diluted sera were incubated with NiV F/G-pseudotyped rVSV for 1 h and then applied to Vero76 cells. After 24 h, luciferase activity was measured. Data shown are neutralization mean ± SD percent values.

To assess the usefulness of our pseudovirus system for PBNA, we evaluated the inhibitory effects of a commercially available polyclonal rabbit anti-NiV-G antibody (pAb-NiV-G) and the sera of NiV-F-immunized mice. Serial dilutions of each sample were incubated with rVSV-NiV-F/G, followed by infection with Vero76 cells and luciferase-based quantification of infectivity. Neutralization was observed only with pAb-NiV-G, which showed an IC_50_ dilution of 3120, whereas NiV-F-immunized mouse serum did not show significant inhibitory activity (Figure 4).

Because a commercial antibody against NiV-F was not available, recombinant protein NiV-F was used to immunize mice and generate anti-NiV-F serum for this assay. Thus, our study focused on validating the pseudovirus-based neutralization system, rather than comparing immunogenicity between F and G antigens.

It should also be noted that the total Ig concentration of the commercial pAb-NiV-G preparation may differ from that of the NiV-F-immunized mouse serum; therefore, the observed differences in neutralization activity reflect a qualitative rather than a quantitative comparison.

This finding suggests that NiV-G plays a more pivotal role in neutralization than NiV-F. Therefore, our results demonstrate that the pseudovirus system developed herein, can be successfully used in PBNA to test NiV-targeted vaccine candidates or therapeutic antibodies.

### 3.5. Development of the In Vivo NiV-F/G-Pseudovirus Infection Mouse Model

Animal models infected with high-risk viruses are essential for evaluating vaccines against high-risk viruses, but implementing these models experimentally presents significant challenges. To determine whether our pseudovirus system can replace high-risk virus infection models, we aimed to develop and quantify a system to infect mice with NiV-F/G-pseudovirus.

**Figure 5:**
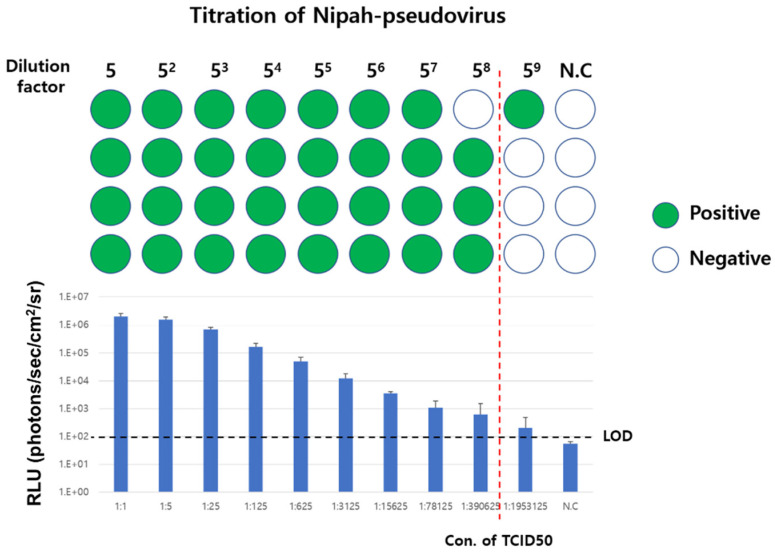
Determination of infectious concentration of NiV F/G-pseudotyped rVSV. Vero76 cells were infected with 5-fold-serially diluted NiV F/G-pseudotyped rVSV. The luciferase signal expressed in each well was quantified 24 h after infection and wells expressing higher signals than the negative control group (i.e., uninfected Vero cells) were determined as ‘infected’ wells. Based on these results, the 50% tissue culture infectious dose (TCID_50_) of NiV F/G-pseudotyped rVSV was determined using the Reed–Muench calculation. In the figure, green circles indicate wells classified as ‘positive’ (infected), whereas empty circles indicate wells classified as ‘negative’ (uninfected).

**Figure 6:**
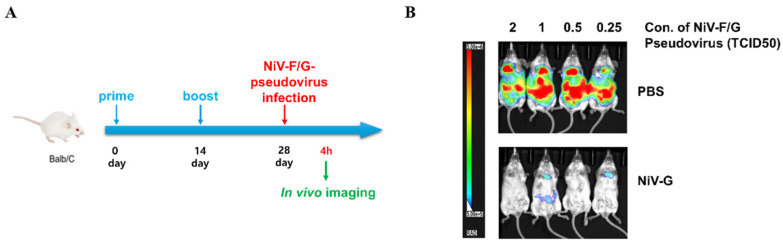
In vivo efficacy verification of vaccine materials using NiV F/G-pseudotyped rVSV. (**A**): Schematic diagram of the in vivo experimental process. Balb/c mice were vaccinated twice with Nipah virus G protein (NiV-G) antigen at 14-day intervals and infected with the NiV F/G-pseudotyped rVSV on day 28. The luminescence signal expressed in the mouse was measured through in vivo imaging at 4 h after infection. (**B**): Temporal distribution of pseudovirus in mice imaged using in vivo imaging system (IVIS). The pseudocolor luminescent signals are overlaid on the grayscale photographic images of mice in the ventral position, with the vertical scale bar indicating bioluminescent radiance (photons/sec/cm^2^/sr). The area where the luminescent signal is expressed indicates infection by pseudovirus.

We first determined the concentration of NiV-F/G-pseudovirus based on cell infectivity. The tissue culture infectious dose 50% (TCID_50_) calculated through the cell infection pattern of NiV-F/G-pseudovirus at each concentration was derived as 2.32 × 10^6^/mL (Figure 5). Subsequently, based on the defined TCID_50_ value, we established an NiV-F/G-pseudovirus infection mouse model and performed an in vivo efficacy evaluation of the NiV vaccine candidate. The data showed that 4 h after infection, NiV-F/G-pseudovirus-infected naïve mice emitted a strong luciferase signal at the site of administration and in the thoracic region, suggesting a strong infection by NiV-F/G-pseudovirus. In contrast, a dramatically reduced luminescence signal was observed in mice vaccinated with the Nipah virus G protein antigen (Figure 6). This result strongly indicates that the glycoprotein of NiV can be used as an efficient vaccine against viral infection and that the pseudovirus infection animal model can be used for the preliminary in vivo evaluation of the efficacy of vaccine candidates.

## 4. Discussion

In this study, we developed a replication-incompetent rVSV pseudotyped with the NiV F and G glycoproteins using a luciferase reporter gene. Our system enables both in vitro and in vivo evaluation of NiV-specific immune responses under BSL-2 conditions and offers a safer and scalable platform for preclinical vaccine assessment.

We determined the optimal F:G plasmid transfection ratio for the production of a pseudovirus and verified an enhancement of transduction efficiency using a luciferase assay. Further, ultracentrifugation and fractionation enabled the enrichment of high-titer pseudovirus fractions, hence providing an effective method for concentration and purification during the preparation of the experimental pseudovirus. Western blot and immunoprecipitation analyses confirmed the incorporation of both the precursor (F0) and cleaved (F1) forms of the NiV F glycoprotein, alongside NiV G, into pseudovirions. These findings indicate proper processing and membrane integration of envelope glycoproteins that are essential for accurately mimicking viral entry and performing robust and efficient neutralization assays [12,13].

Syncytia formation during the production of the pseudovirus was also observed. Co-transfection with NiV-F and NiV-G resulted in pronounced syncytia formation in pCAGGS-NiV-F/G-transfected cells, whereas the control groups (vector only, F only, and G only) did not exhibit any syncytia formation (Figure 1B). These observations suggest that the co-expression of both NiV-F and NiV-G is required for effective production of the pseudovirus. This requirement is consistent with the well-characterized mechanism at play in henipaviruses, in which case, NiV-G binding to ephrin-B2/B3 triggers the conformational activation of NiV-F to mediate membrane fusion and syncytia formation, whereas the expression of either glycoprotein alone fails to produce CPE [14,15].

Importantly, our experiments served as a proof-of-principle for the applicability of this system in real-time in vivo imaging. Specifically, using a luciferase-based bioluminescence imaging platform, we established a mouse model in which NiV-F/G-pseudotyped rVSV was administered intraperitoneally to visualize infection. Strong luminescent signals were observed at the injection site and in the thoracic region of non-immunized mice, whereas vaccinated animals immunized with the recombinant NiV-G protein, which has been proposed as a promising target antigen for vaccines to prevent Nipah virus infection [16,17,18], showed significantly reduced luminescence. And, although statistically limited, our study also demonstrated that NiV-G antigen effectively induced protective immunity (Figure 6). These results confirm the applicability of our pseudovirus system for evaluating vaccine-mediated protection in vivo and are consistent with the potential protective efficacy of NiV-G suggested by previous studies [19,20]. This dual in vitro and in vivo evaluation strongly supports the usefulness of the G protein as a promising target for NiV vaccine design and justifies other pandemic-preparedness agendas using the systems reported herein.

The proposed rVSV-based model shows several advantages. The VSV genome replicates and is transcribed exclusively in the cytoplasm, with an mRNA half-life of only a few minutes, thus driving robust luciferase or fluorescent protein expression detectable as early as 2–6 h post-infection [21,22]. In turn, this enables rapid, single-day neutralization or entry-inhibition assays, in contrast to lentiviral pseudotypes, which typically require 48–72 h to reach assay endpoints [6,21,23]. Additionally, it is known that Nipah virus infection primarily begins in respiratory and lung epithelial cells, and then, as the infection progresses, enters the bloodstream and spreads to various organs, including the brain [24]. In our current study (Figure 6), the observation of a strong signal in the thoracic region by intraperitoneally administered Nipah pseudovirus suggests that pseudovirus systems can mimic the physiological infection pattern of authentic Nipah virus. At the same time, the strong signal at the pseudovirus injection site suggests that the infection can be preferentially induced at the injection site, implying the possibility of establishing an organ-specific infection model using pseudovirus.

Furthermore, ΔG pseudotyped viruses are replication-defective, as they undergo only one round of infection because progeny virions lack envelope proteins. This feature allows studies involving high-risk viral glycoproteins, such as those from Ebola, Nipah, MERS-CoV, and SARS-CoV-2, to be performed under BSL-2 conditions, thereby overcoming infrastructure barriers and enabling broader collaborative research.

While ΔG-pseudotyped VSV particles have been explored for in vitro entry and neutralization assays, reports on their use for real-time in vivo imaging of protective efficacy in small animal models have remained limited. Our findings fill this gap by evaluating a non-replicating BSL-2-compatible pseudovirus-based infection model that enables visual monitoring of vaccine-induced protection in vivo.

Despite these strengths, this platform shows some limitations. Foremost, the use of recombinant VSV-expressed proteins NiV-F and NiV-G and antibodies raised against recombinant antigens is an important limitation of our study, as, despite sequence identity, such systems may not fully reproduce the post-translational modifications, conformational dynamics, and immunogenic properties of natural Nipah virus proteins or antibodies elicited by natural infections. Hence, future verification using authentic Nipah virus isolates and sera from convalescent patients would lend decisive support to the findings reported herein. In addition, as non-replicating viruses, pseudoviruses do not recapitulate systemic viral replication, dissemination, or pathogenesis. In addition, the platform primarily captures humoral immune responses and may not fully account for the contributions of T cell-mediated immunity, which is important in the context of NiV protection. Finally, although common, the use of Vero76 cells for neutralization assays may not reflect the full range of host-specific receptor interactions.

Nevertheless, the NiV F/G-pseudotyped rVSV system described herein provides a novel, robust, and versatile tool for evaluating neutralizing antibodies and vaccine candidates under accessible biosafety conditions. In particular, its ability to enable both in vitro analysis and in vivo bioluminescence imaging offers new opportunities for rapid and safe preclinical assessment of novel immunogens and may be readily adapted for other emerging high-containment pathogens.

## 5. Conclusions

We established a VSV-based pseudovirus system capable of quantifying infections and vaccine-mediated protection in vitro and in vivo. This new platform bridges the gap between traditional in vitro neutralization assays and live virus-challenge models, hence contributing to the development of safe and scalable tools for vaccine-related research on NiV and other emerging BSL-4 pathogens.

## Data Availability

The raw data supporting the conclusions of this article will be made available by the authors on request.

## Abbreviations

The following abbreviations are used in this manuscript:

NiV	Nipah virus
rVSV	Recombinant vesicular stomatitis virus
F	Fusion glycoprotein
G	Attachment glycoprotein
VSV	Vesicular Stomatitis Virus
PBNA	Pseudovirus-based neutralization assay
IP	Immunoprecipitation
HRP	Horseradish peroxidase
IC50	50% inhibitory concentration
TCID50	Tissue culture infectious dose 50%
RLU	relative light units
pAb-NiV-G	polyclonal rabbit anti-NiV-G antibody
